# Cross-sectional survey of depressive symptoms and suicide-related ideation at a Japanese national university during the COVID-19 stay-home order

**DOI:** 10.1186/s12199-021-00953-1

**Published:** 2021-03-05

**Authors:** Kyoko Nomura, Sachiko Minamizono, Eri Maeda, Roseline Kim, Toyoto Iwata, Junko Hirayama, Kyoichi Ono, Masahito Fushimi, Takeshi Goto, Kazuo Mishima, Fumio Yamamoto

**Affiliations:** 1 Department of Environmental Health Science and Public Health, Akita University Graduate School of Medicine, Akita, Japan; 2 Department of Cell Physiology, Akita University Graduate School of Medicine, Akita, Japan; 3 Akita University, Akita, Japan; 4 Department of Neuropsychiatry, Akita University Graduate School of Medicine, Akita, Japan

**Keywords:** COVID-19 pandemic, Depressive symptoms, Stay-home order, Suicide-related ideation, University students

## Abstract

**Background:**

We aimed to estimate the prevalence of depressive symptoms as well as suicide-related ideation among Japanese university students during the stay-home order necessitated by the coronavirus disease 2019 pandemic in Japan, and offer evidence in support of future intervention to depression and suicide prevention strategies among college and university students.

**Methods:**

The data for this cross-sectional study were derived from the Student Mental Health Survey conducted from May 20 to June 16, 2020 at a national university in Akita prefecture. Among the 5111 students recruited, 2712 participated in this study (response rate, 53%; mean age ± standard deviation, 20.5 ±3.5 years; men, 53.8%). Depressive symptoms were identified by using the Patient Health Questionnaire-9 (PHQ-9).

**Results:**

The prevalence of moderate depressive symptoms based on a PHQ-9 score ≥10 and suicide-related ideation based on question 9 of PHQ-9 ≥1, which encompasses thoughts of both suicide and self-harm, was 11.7% and 6.7%, respectively. Multivariable logistic regression analyses showed that risk factors for depression included being a woman, smoking, alcohol consumption, and social network communication using either video or voice. For suicide-related ideation, alcohol consumption was the only risk factor. Exercise and having someone to consult about worries were associated with decreased risk of both depressive symptoms and suicide-related ideation.

**Conclusions:**

Negative lifestyles of smoking and drinking, and being a woman, may be important risk factors for depressive symptoms, whereas exercise and having someone to consult about worries may be protective factors.

**Supplementary Information:**

The online version contains supplementary material available at 10.1186/s12199-021-00953-1.

## Introduction

The outbreak of the infectious coronavirus disease 2019 (COVID-19) was first reported in Wuhan, China, in December 2019, subsequently spreading to the extent of becoming a global pandemic. In Japan, the first case of COVID-19 was confirmed on January 15, 2020, and in March [1], the number of new cases sharply increased. In order to limit both the spread of the virus and overwhelming demands for medical care, on April 16, Japanese Prime Minister declared a nationwide state of emergency. Among the special measures against COVID-19 in Japan, the governor of each prefecture is empowered to call on residents to stay at home (self-quarantine) and refrain from going out. However, owing to the Japanese Constitution’s emphasis on the protection of citizens’ rights, a forced lockdown of cities through the imposition of fines on people going out without permission, as implemented in parts of Europe and the USA, was not possible. Nevertheless, educational institutions, restaurants, and public facilities were all closed, leaving cities and towns deserted.

Regarding the health consequences of the pandemic and subsequent isolation measures, depression is one of the most serious as it is closely linked with suicide. In Japan, according to Japanese vital statistics, in 2017, suicide was the first leading cause of death among individuals aged between 10 and 39, and the country’s suicide rate is the highest among all G7 members [2]. Early evidence suggests the massive impact of lockdowns on psychological distress, with reports of high prevalence rates of depression, anxiety, and sleep disturbance [3–6]. A study in Belgium [7] reported that during the lockdown, young adults experienced a greater level of depression than their older counterparts, which may be explained by an intolerance of uncertainty. An Italian study [8] reported that the impact of the lockdown on psychological distress was greater in students than in workers. Thus, the health consequences of disasters differ by population subgroups, and it is clear that young adolescents are among the most vulnerable. Furthermore, given the high suicide rate in the younger generation in Japan, the psychological distress brought on by the COVID-19 pandemic may have been significant enough to affect students’ mental health.

The rationale behind the closure of educational institutions was that young adolescents could contract COVID-19 and, owing to the high possibility of being asymptomatic, unknowingly carry and transmit the virus to the community. In this situation, the stay-home order left students who had traveled far from their hometowns to attend college and who were, therefore, alone in unfamiliar cities, completely alone. This was especially true for freshers, who were forbidden even to visit school, and had not yet had the opportunity to get to know their fellow students, resulting in temporary social withdrawal. Given the lack of Japan-specific data, with the aim of providing a reference for campus psychological services, this study sought to investigate depressive symptoms and suicide-related ideation in university students under the stay-home order and determine the risk and protective factors.

## Methods

### Participants

This cross-sectional study was a part of the Student Mental Health Survey that was conducted at Akita University between May 20 and June 16, 2020. As of May 16, 2020, 5111 graduate and undergraduate students were enrolled in Akita University. In Akita prefecture, while the self-quarantine period began on April 26 and officially ended on May 31, in reality, it continued until June 19 because all residents were strongly advised to refrain from going out or interacting with anyone, including family members, from beyond the boundary of the prefecture. Akita University contacted all students either by email or phone and confirmed that the majority were staying home. A very small number, however, had returned to their hometowns for urgent reasons (e.g., family health issues).

All students were approached via institutional emails and asked to log in to the e-classroom platform, where they would find a link to the online self-administered questionnaire. The first reminder for those who had not responded yet was sent 2 weeks after the initial email. Of the 2712 students enrolled in the study (response rate 53%), we excluded those who had missing values on the Patient Health Questionnaire (PHQ-9, *n*=257). Accordingly, the data of 2449 students were analyzed.

This study was approved by the Institutional Review Board of Akita University Medical School (No. 2520). On the first page of the online self-administered questionnaire, students were given an explanation of the purpose of the study, the fact that participation was entirely voluntary, that they could withdraw from the study at any time without any repercussions with regard to their academic records, and that their data would be kept confidential. Subsequently, only interested students began answering the questionnaire. They were also provided with the opportunity to submit an opt-out withdrawal form, provided on the office of research administration website.

### Questionnaire

A web-based survey composed of 51 multiple-choice questions was launched on May 20 and remained open until June 16. The survey, which took approximately 15 min to complete, included questions on living arrangement (alone or living with family/others), hometown (within Akita or outside Akita), the presence of someone to consult about worries, smoking status (never, former, current), alcohol consumption (6–7 days/week, 3–4 days/week, 1–2 days/week, never), daily exercise (min per day), frequency of communication with people in their social networks (6–7 days/week, 3–4 days/week, 1–2 days/week, never), people they communicated with (family, friends, boy/girlfriends, acquaintances, strangers), the frequency of leaving the house for essential purposes, and worries about financial strain, academic attainment, health, social activity, and social support. Participants were asked to indicate which of the abovementioned five domains of worry they were most concerned about. Daily exercise was measured according to intensity—light (up to 4 metabolic equivalents (METS)), moderate (5–6 METS), vigorous (7–8 METS), and very vigorous (9–10 METS)—and multiplied with exercise time period and divided into quartiles (highest, second highest, second lowest, lowest). For communication with people in their social networks, the avenues considered were text (e.g., LINE, Twitter, Facebook), voice (e.g., telephone, iPhone, mobile phone, LINE), and video (e.g., Skype, LINE, ZOOM).

### PHQ-9

Depressive symptoms were identified with the validated Japanese version of the PHQ-9 [9, 10], which was based on the nine criteria for depression proposed by the Diagnostic and Statistical Manual of Mental Disorders, 5th edition. Each item was rated on a four-point Likert scale ranging from 0 (not at all) to 3 (almost every day). PHQ-9 scores were divided into five groups representing varying levels of severity of depressive symptoms: 0–4 (minimal or none), 5–9 (mild), 10–14 (moderate), 15–19 (moderately severe), and 20–27 (severe). The total score ranged from 0 to 27, and the higher the score, the more intense the depressive symptoms. Reliability, as depicted by Cronbach’s alpha, was 0.86. The established PHQ-9 cutoff score of 10 (PHQ-9 ≥10), which has previously demonstrated high sensitivity and specificity in detecting major depression was used [11, 12].

For suicide-related ideation, question 9 of the PHQ-9, which encompasses thoughts of both suicide and self-harm [13], was used referring to previous literatures [14, 15]. Participants were asked “Have you thought that you would be better off dead or of hurting yourself in some way?” The response received 0 for none, 1 for at least 2 days per week, 2 for at least 1 week, and 3 for nearly every day. Thus, the score of question 9 ≥1 was considered indicative of suicide-related ideation. In our analyses, the score of question 9 ≥1 was treated as “suicide-related ideation” and the score of question 9 ≥2 was treated as “severe suicide-related ideation.”

### Statistical analysis

First, we estimated the prevalence and 95% confidence intervals (CIs) of depressive symptoms including suicide-related ideation. Second, we conducted bivariable *χ*2 analysis to assess the association between demographic characteristics and depressive symptoms. Third, we used logistic regression to estimate odds ratios (ORs) and 95% CIs for the association between each sociodemographic factor and depressive symptoms. Adjusting for covariates investigated in univariable models, multivariable logistic regression modeling was used to evaluate risk and protective factors for depressive symptoms. The statistical interaction between gender and other covariates was investigated.

All analyses were performed using STATA14-MP (Stata Corp LP, College Station, TX, USA). A two-sided *p* value of < 0.05 was considered statistically significant.

## Results

Demographic characteristics were shown in Table 1. Of the 2449 students (mean age, 20±2 years) included in the analyses, 757 (42% women) were freshers, 504 (51% women) were sophomores, 479 (51% women) were juniors, 334 (53% women) were seniors, 226 (30% women) were graduate students, and 129 (50% women) were classified as “others.” The majority (86%) of 1419 students who came from other prefectures lived alone. In total, 22% of the sample reported that they did not have anyone with whom they could discuss their worries, but this was more common in men than in women (see Table 1).

**Table 1:** Baseline characteristics of enrolled students (*n*=2449)

	Men (*n*=1308)		Women (*n*=1119)		*p*
	*n*	%	*n*	%	
Age, mean (sd)	20 (2)		20 (2)		0.386
Hometown					<0.001
Outside Akita	858	66	570	51	
Within Akita	450	34	549	49	
Living alone					<0.001
Alone	874	68	593	54	
Not alone	420	32	513	46	
Exercise					<0.001
Highest quartile	339	29	206	20	
Second highest quartile	280	24	266	26	
Second lowest quartile	270	23	282	28	
Lowest quartile	280	24	256	25	
Smoking					<0.001
Current	60	5	11	1	
Past	45	3	13	1	
Never	1204	92	1093	98	
Alcohol					<0.001
5-7/week	33	3	13	1	
3-4/week	60	5	28	3	
1-2/week	280	21	199	18	
Never-seldom	931	71	877	79	
Social network service daily use					
Text message	1101	84	1052	94	<0.001
Sound	204	16	197	18	0.182
Video	68	5	66	6	0.444
Either sound or video	231	18	217	19	0.264
Worries					<0.001
Financial strain	298	23	224	20	
Academic record	313	24	253	23	
Leisure	355	27	325	29	
Social support	137	11	218	19	
Physical activity	195	15	101	9	
Anyone to consult					<0.001
Yes	957	73	934	83	
None	346	27	187	17	

The sum of the category that does not reach 2429 indicates missing values

Prevalence of depressive symptoms and suicide-related ideation is shown in Table 2. Median with interquartile range of PHQ-9 was 3 with 0-6 in total, 2 with 0-6 in men, and 3 with 1-6 in women. In total, 11.6% of the sample scored ≥10 on the PHQ-9, which was indicative of depressive symptoms (11.2% of men; 12.0% of women) and 6.7% scored ≥1 on question 9 of the PHQ-9 (6.7% of men; 6.6% of women), which was indicative of suicide-related ideation. Men were more likely to have no depressive symptoms, whereas women were more likely to experience mild depressive symptoms.

**Table 2:** Prevalence of depressive symptoms and suicide related ideation

	All (*n*=2449)		Male (*n*=1309)		Female (*n*=1121)	
	*n* (%)	95% CI	*n* (%)	95% CI	*n* (%)	95% CI
PHQ-9						
None (0-4)	1602 (65.4%)	63.5-67.3%	904 (69.1%)	66.5-71.6%	682 (60.8%)	57.9-63.7%
Mild (5-9)	564 (23.0%)	21.3-24.8%	258 (20.0%)	17.6-22.0%	304 (27.1%)	24.5-29.8%
Moderate (10-14)	191 (7.8%)	6.8-8.9%	98 (7.5%)	6.1-9.1%	93 (8.3%)	6.8-10.1%
Moderately severe (15-19)	57 (2.3%)	1.8-3.0%	30 (2.3%)	1.6-3.3%	26 (2.3%)	1.5-3.4%
Severe (≥20)	35 (1.4%)	1.0-2.0%	19 (1.5%)	0.9-2.3%	16 (1.4%)	0.8-2.3%
Moderate~severe (10−)	283 (11.6%)	10.3-12.9%	147 (11.2%)	9.6-13.1%	135 (12.0%)	10.2-14.1%
Suicide-related ideation	162 (6.7%)	5.7-7.7%	88 (6.7%)	5.4-8.2%	73 (6.6%)	5.2-8.2%
Severe suicide-related ideation	45 (1.9%)	1.4-2.5%	21 (1.6%)	1.0-2.5%	24 (2.2%)	1.4-3.2%

Risk and protective factors for depressive symptoms are shown in Table 3. The factors significantly associated with a PHQ-9 score ≥10 included being aged 20 years or older (*p*=0.007), coming from other prefectures (*p*=0.004), living alone (*p*=0.002), low-intensity physical exercise (*p*<0.001), being a current smoker (*p*<0.001), frequent alcohol consumption (*p*<0.001), social network communication using either sound or video (*p*<0.001), worries about academic record and social support (*p*<0.001), and having no one to consult about worries (*p*<0.001). The factors associated with a score ≥1 on item 9 of the PHQ-9 were low-intensity physical exercise (*p*=0.006) and greater alcohol consumption (*p*<0.001). Worries and having no one to consult were statistically associated with suicide-related ideation (*p*=0.003 and *p*<0.001, respectively).

**Table 3:** Risk and protective factors for depressive symptoms

	Moderate-severe level depression				*p*	Suicide-related ideation				*p*	Severe suicide-related ideation				*p*
	(+)		(−)			(+)		(−)			(+)		(−)		
	*n*	%	*n*	%		*n*	%	*n*	%		*n*	%	*n*	%	
Sex					0.533					0.865					0.318
Women	135	48	986	46		73	45	1038	46		24	53	1087	46	
Men	147	52	1162	54		88	55	1217	54		21	47	1284	54	
Age					0.009					0.138					0.060
<20	100	35	943	44		60	37	975	43		13	29	1022	43	
20−	182	65	1218	56		102	63	1292	57		32	71	1362	57	
Hometown					0.004					0.226					0.642
Outside Akita	189	67	1248	58		88	54	1342	59		28	62	1402	59	
Within Akita	94	33	913	42		74	46	926	41		17	38	983	41	
Living alone					0.002					0.745					0.505
Alone	193	70	1281	60		99	62	1368	61		29	66	1438	61	
Not alone	84	30	859	40		60	38	876	39		15	34	921	39	
Exercise					<.001					0.006					0.011
Highest quartile	49	19	502	26		28	19	484	24		7	17	541	25	
Second highest quartile	54	21	497	26		30	21	518	25		9	22	541	25	
Second lowest quartile	62	24	492	25		34	23	520	25		6	15	546	25	
Lowest quartile	93	36	447	23		53	37	520	25		19	46	518	24	
Smoking					<.001					0.009					0.003
Current	19	7	52	2		9	6	62	3		5	11	66	3	
Past	12	4	46	2		8	5	50	2		2	4	56	2	
Never	252	89	2062	95		145	90	2155	95		38	84	2262	95	
Alcohol					<.001					<.001					<0.001
5-7/week	13	5	33	2		9	6	37	2		4	9	42	2	
3-4/week	16	6	73	3		11	7	77	3		6	13	82	3	
1-2/week	62	22	419	19		28	17	452	20		7	16	473	20	
Never~seldom	192	68	1631	76		114	70	1697	75		28	62	1783	75	
Social network service daily use															
Either sound or video	73	26	379	18	<.001	37	23	411	18	0.1339	12	27	436	18	0.150
Worries					<.001					0.0025					0.303
Financial strain	63	22	465	22		39	24	487	22		10	23	516	22	
Academic record	80	28	492	23		43	27	526	23		12	27	557	23	
Leisure	56	20	628	29		36	22	642	28		10	23	668	28	
Social support	56	20	301	14		35	22	321	14		10	23	346	15	
Physical activity	26	9	270	13		8	5	287	13		2	5	293	12	
Anyone to consult about worries					<.001					<.001					<0.001
Yes	148	52	1760	81		75	46	1821	80		24	53	1872	79	
None	135	48	400	19		87	54	446	20		21	47	512	21	

**Fig. 1:**
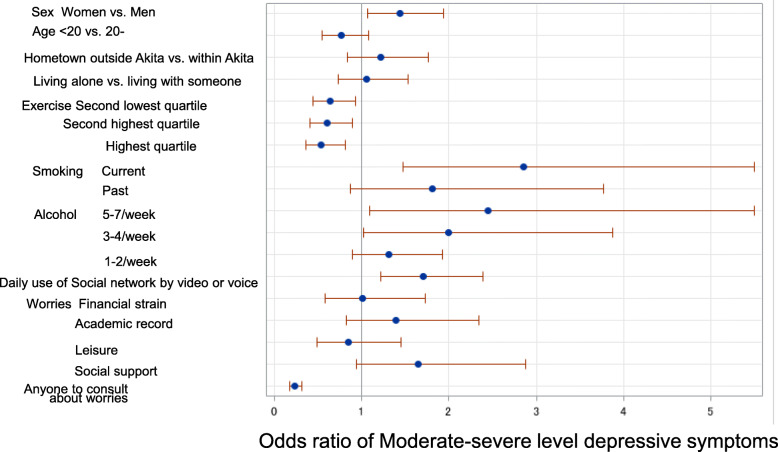
Factors associated with moderate-severe level depression

Multivariable logistic regression analyses are shown in Fig. 1. Multivariable logistic regression analyses showed that being a woman (OR 1.45, 95% CI, 1.07–1.94), current smoking (OR 2.85, 95% CI, 1.48–5.50), weekly alcohol consumption frequency of 5–7 times (OR 2.45, 95% CI, 1.09–5.50) and 3–4 times (OR 1.99, 95% CI, 1.02–3.88), and daily social network communication using either voice or video (OR 1.71, 95% CI, 1.22–2.40) were associated with an increased risk of depressive symptoms. Protective factors included exercise (highest quartile OR 0.54, 95% CI, 0.36–0.81; second highest quartile OR 0.60, 95% CI, 0.41–0.89; second lowest quartile OR 0.64, 95% CI, 0.44–0.93, trend *p*=0.002) and having someone to consult about worries (OR 0.24, 95% CI, 0.18–0.32). Statistical interaction was not observed between gender and any other covariates. Multivariable logistic regression models for the risk of suicide-related ideation are shown in a supplementary table. The risk factors included alcohol consumption at a weekly frequency of 5–7 times (OR 2.60, 95% CI, 1.03–6.55) and 3–4 times (OR 2.51, 95% CI, 1.18–5.37) and worries (i.e., financial strain OR 2.79, 95% CI, 1.13-6.89, academic record OR 3.05, 95% CI, 1.25-7.44, social support OR 4.36, 95% CI, 1.73-10.97 compared to physical activity). In contrast, the protective factors included exercise (highest quartile, OR 0.59, 95% CI, 0.36–0.99), outside Akita (OR 0.61, 95% CI, 0.38-0.96), and having someone to consult about worries (OR 0.20, 95% CI, 0.14–0.29). Statistical interaction was not observed between gender and any other covariates.

## Discussion

Although Japan did not enforce a strict lockdown like other countries, we still found that university students experienced psychological burden, with approximately 11% manifesting a moderate level of depressive symptoms, identified by a PHQ-9 score ≥10. Negative lifestyles such as smoking and drinking, and being a woman, may be important risk factors for depressive symptoms, while exercise and having someone to consult about worries may be protective factors.

The prevalence of depression among college and university students under the COVID-19 pandemic varies depending on the diagnostic tools utilized, population in question, duration of quarantine/isolation, and how severely the areas in question have been affected [16]. However, in previous studies of college and university students using the PHQ-9, the following were the depression prevalence (PHQ-9 ≥10) rates reported: 46.5% in the UK [17], 9.0% in China [18], 18.5% in Slovakia [19], and 31.7% in Ukraine [20]. Although a direct comparison between countries is difficult, within Japan, a study [14] investigating the pre-pandemic situation among 2194 students at one national university reported a 28.7% prevalence of depression (95% CI, 27–31) based on PHQ-9 scores ≥5. With the same diagnostic cutoff point (PHQ-9 ≥5), the prevalence of depression among our population reached 34% (95% CI, 33–37), indicating a statistically higher prevalence [14]. Thus, the stay-home order appears to have been detrimental to students’ psychological health.

In our study, we found that mild to moderate levels of depressive symptoms were more common in women than in men, but these differences disappeared with moderately severe to severe depressive symptoms and suicidal ideation. A meta-analysis of studies based on nationally representative samples in the USA suggested that generally, females have a high risk of depression; however, the higher depression prevalence in females than in males was observed only in early adolescence and gradually disappeared in adulthood [21]. A US study [22] on 1344 college students aged 18–29 years (12% Asian) identified women are more likely to develop depressive symptoms (OR 1.99, 95%CI, 1.07–2.90). However, this difference between genders disappeared with suicidal ideation which was consistent with our results. Among the few studies investigating suicidal ideation in college students globally, a study [23] on 33,635 students in grades 7–12 (13–18 years) in Beijing, China reported that the prevalence of suicidal ideation was significantly higher in girls (13.3%) than boys (10.7%). The difference between the study [23] and ours is probably attributed to the age differences of the samples in the two studies. While the mechanisms of higher depression prevalence in females remain unclear, the interplay of gender socialization, social and hormonal mechanisms, and stressful events associated with adolescence may play a role [24]. Alternatively, the absence of gender difference in suicidal ideation may be explained by the differences favoring men in suicide rates, where the rates among males were about three times those among females in Japan [25].

The present study demonstrated that unfavorable lifestyle habits, such as smoking and alcohol consumption, were associated with depressive symptoms. In a meta-analysis [26], nearly half of the 148 studies reported that baseline depression was associated with later smoking behavior, while over a third found evidence that exposure to smoking was associated with later depression. Although the direction mechanism of the relationship between smoking and depression is not clear, a causal mechanism usually requires a certain exposure period to cause illness. This may not have been possible in our young sample, where nearly half of the participants were under the age of 20 and therefore, prohibited by law from smoking or consuming alcohol. Hence, a reasonable interpretation of our findings may be that individuals started smoking or drinking after the onset of depressive symptoms. Indeed, depressive status is associated with increased vulnerability to substance use. The relationship between alcohol consumption and depressive symptoms in our study became stronger as weekly alcohol intake increased. It is well established that there are strong links between heavy alcohol consumption and mental health problems [27]. In addition, a study [28] reported that a reduced-nicotine standard for cigarettes may reduce smoking without worsening depressive symptoms, which is consistent with the results of longitudinal studies. This may suggest that quitting smoking leads to decreased recurrence of depression [29]. Future studies should seek to explore the reduction of smoking and alcohol consumption as a way to identify potential causal pathways between unfavorable lifestyles and depression.

In contrast, a favorable lifestyle choice—engaging in exercise—was associated with a decreased risk of depressive symptoms, with a dose-response relationship. This finding requires careful interpretation because in this cross-sectional study, we simultaneously investigated exposure and outcome. Students with depressive symptoms may refrain from engaging in exercise even though it can improve their psychological status. Nevertheless, a meta-analysis of 51 randomized controlled trials [30] reported that exercise may be an effective therapy for treating depression in university students, while another meta-analysis [31] suggested that exercise is a promising treatment for depression in adults, showing effects that are comparable to first-line treatments.

In the present study, it was found that daily social network communication using either video or voice was associated with depressive symptoms. The majority of the students who used video or voice features every day contacted their family, friends, and acquaintances, but 60 students used social networks for communication with unspecified people. Connecting with strangers on social networking platforms may be problematic because it does not involve the responsibility required in relationships, potentially leading to irresponsible and hurtful behavior [32]. In young users, this can be detrimental to academic achievement [33]. Consequently, these people may reduce their community participation, eventually becoming socially withdrawn, who are accompanied with psychomorbidity [34]. A study [35] that investigated 5972 students randomly selected from six universities in China demonstrated that poor social contact was significantly associated with an increased risk of suicidal ideation. We performed an additional analysis to determine if the 60 students connecting with strangers displayed an association with increased risk of suicide-related ideation but found no such trend. Although we were unable to determine whether daily use of video or voice communication tools was associated with internet addiction, in a previous study, internet addiction was significantly associated with psychiatric comorbidity, including depression [36]. Further, a meta-analysis provides evidence that internet addiction is associated with increased suicidality even after adjusting for potential confounding variables including depression [37]. Nevertheless, the importance of internet addiction in this area of research needs further exploration. Although the cross-sectional design of this study posed a barrier to the determination of causality, the relationship between social network use (whether video or voice) and depression could be considered indicative of the fact that loneliness drives individuals to the active use of communication tools.

In our study, the presence of someone to consult about worries was associated with a decreased risk of depressive symptoms and suicide-related ideation. There is an abundance of evidence demonstrating that social support plays a major role in alleviating psychological distress. Factors such as family cohesion and connections with friends can play a protective role against suicidal behavior [38]. In our study, approximately 27% of men and 17% of women answered that they did not have anyone to consult about their worries. Help-seeking behavior, a powerful coping skill with regard to mental illness, can be difficult to learn [39]. Thus, there is a need for school intervention programs that cover protective skills in addition to self-care and social support from teachers, friends, and health or educational professionals.

Despite its strengths, this study has several limitations that need to be addressed. First, as the setting was a single university in Japan, the findings have limited generalizability. However, this limitation may be countered by the fact that the response rate exceeded 50% and the sample size was large. Second, the prevalence of depressive symptoms might have been underestimated because participation was voluntary. For example, students with depressive symptoms may have found it difficult to answer a 51-item questionnaire. In addition, some students may have hesitated to share their health information with the university despite the fact that we repeatedly explained that the purpose of the study was to screen high-risk individuals for prompt intervention. Third, owing to Japan’s relatively low fatality rate, depressive symptoms may not have been very severe. In addition, even though a prefectural governor issued the request for people to stay indoors, they were still allowed to go out for essential purposes such as seeking medical care, purchasing necessary supplies, or commuting to an essential job. If the COVID-19 situation in Japan worsens, the severity and frequency of depressive symptoms may become more apparent. Fourth, because 85% of those who came from prefectures outside Akita lived alone, these two variables were highly correlated. To identify the problem of collinearity in multivariable regression analyses, we excluded those who lived alone or whose hometowns were outside Akita, but the results did not change. Finally, owing to the cross-sectional design, we were unable to make causal inferences regarding the relationships between variables.

## Conclusion

We investigated depressive symptoms as well as suicide-related ideation among Japanese university students during the COVID-19 stay-home order and found that negative lifestyles of smoking and drinking, and being a woman, may be important risk factors for depressive symptoms, whereas exercise and having someone to consult about worries may be protective factors.

## Supplementary Information

**Additional file 1:.** sTable 1. Factors associated with suicide-related ideation.

## Data Availability

The datasets used and/or analyzed during the present study are available from the corresponding author on reasonable request.

## Abbreviations

COVID-19: Coronavirus disease 2019
METS: Metabolic equivalents
PHQ-9: Patient Health Questionnaire-9
OR: Odds ratio
CI: Confidence interval
