# Supplementary material for: Cross-sectional survey of depressive symptoms and suicide-related ideation at a Japanese national university during the COVID-19 stay-home order
Source: Environ Health Prev Med. 2021 Mar 5;26:30. doi: 10.1186/s12199-021-00953-1 (PMC7934991; doi:10.1186/s12199-021-00953-1)
Supplement: Supplementary file 1 — Additional file 1:. sTable 1. Factors associated with suicide-related ideation. [file 12199_2021_953_MOESM1_ESM.docx]

sTable 1. Factors associated with suicide-related ideation

|  |  | Suicide-related ideation | | | Severe suicide-related ideation | | |
| --- | --- | --- | --- | --- | --- | --- | --- |
|  |  | Adjusted　OR | 95%CI | | Adjusted　OR | 95%CI | |
|  |  |  | Lower | Upper |  | Lower | Upper |
| Hometown | |  |  |  |  |  |  |
|  | Outside Akita | 0.61 | 0.38 | 0.96 | 1.20 | 0.50 | 2.85 |
|  | Within Akita | 1.00 | ‐ | ‐ | 1.00 | ‐ | ‐ |
| Exercise | |  |  |  |  |  |  |
|  | Highest quartile | 0.59 | 0.36 | 0.99 | 0.42 | 0.17 | 1.06 |
|  | Second highest quartile | 0.65 | 0.40 | 1.07 | 0.57 | 0.25 | 1.34 |
|  | Second lowest quartile | 0.67 | 0.41 | 1.07 | 0.36 | 0.14 | 0.94 |
|  | Lowest quartile | 1.00 | ‐ | ‐ | 1.00 | ‐ | ‐ |
| Smoking | |  |  |  |  |  |  |
|  | Current | 1.92 | 0.82 | 4.50 | 3.36 | 1.06 | 10.68 |
|  | Past | 2.01 | 0.85 | 4.75 | 1.63 | 0.34 | 7.78 |
|  | Never | 1.00 | ‐ | ‐ | 1.00 | ‐ | ‐ |
| Alcohol | |  |  |  |  |  |  |
|  | 5-7/week | 2.60 | 1.03 | 6.55 | 2.69 | 0.64 | 11.34 |
|  | 3-4/week | 2.51 | 1.18 | 5.37 | 4.65 | 1.58 | 13.67 |
|  | 1-2/week | 0.88 | 0.52 | 1.48 | 0.97 | 0.38 | 2.51 |
|  | Never~seldom | 1.00 | ‐ | ‐ | 1.00 | ‐ | ‐ |
| Worries | |  |  |  |  |  |  |
|  | Financial strain | 2.79 | 1.13 | 6.89 | 1.59 | 0.33 | 7.78 |
|  | Academic record | 3.05 | 1.25 | 7.44 | 2.30 | 0.50 | 10.67 |
|  | Leisure | 2.27 | 0.92 | 5.61 | 1.69 | 0.35 | 8.14 |
|  | Social support | 4.36 | 1.73 | 10.97 | 3.37 | 0.70 | 16.29 |
|  | Physical activity | 1.00 | ‐ | ‐ | 1.00 | ‐ | ‐ |
| Anyone to consult about worries | | |  |  |  |  |  |
|  | Yes | 0.20 | 0.14 | 0.29 | 0.33 | 0.17 | 0.65 |
|  | None | 1.00 | ‐ | ‐ | 1.00 | ‐ | ‐ |
